# Cholesterolomics: An update

**DOI:** 10.1016/j.ab.2017.01.009

**Published:** 2017-05-01

**Authors:** William J. Griffiths, Jonas Abdel-Khalik, Eylan Yutuc, Alwena H. Morgan, Ian Gilmore, Thomas Hearn, Yuqin Wang

**Affiliations:** Swansea University Medical School, Singleton Park, Swansea SA2 8PP, UK

**Keywords:** Cholesterol, Oxysterols, Cholestenoic acids, Derivatisation, Mass spectrometry

## Abstract

Cholesterolomics can be regarded as the identification and quantification of cholesterol, its precursors post squalene, and metabolites of cholesterol and of its precursors, in a biological sample. These molecules include 1,25-dihydroxyvitamin D_3_, steroid hormones and bile acids and intermediates in their respective biosynthetic pathways. In this short article we will concentrate our attention on intermediates in bile acid biosynthesis pathways, in particular oxysterols and cholestenoic acids. These molecular classes are implicated in the aetiology of a diverse array of diseases including autoimmune disease, Parkinson's disease, motor neuron disease, breast cancer, the lysosomal storage disease Niemann-Pick type C and the autosomal recessive disorder Smith-Lemli-Opitz syndrome. Mass spectrometry (MS) is the dominant technology for sterol analysis including both gas-chromatography (GC)-MS and liquid chromatography (LC)-MS and more recently matrix-assisted laser desorption/ionisation (MALDI)-MS for tissue imaging studies. Here we will discuss exciting biological findings and recent analytical improvements.

## 1 Introduction

Gas chromatography – mass spectrometry (GC-MS) with the utilisation of stable-isotope labelled standards is the gold standard methodology for the analysis of neutral cholesterol metabolites [1], [2], [3]. Acidic metabolites are efficiently analysed by liquid chromatography (LC)-MS, where optimal analysis is similarly performed with the use of isotope labelled standards [4], [5]. GC-MS with appropriate derivatisation is also suitable for analysis of acidic metabolites [6], while LC-MS can also be used for the analysis of neutrals with appropriate ionisation additives or derivatisation methods [7], [8], [9], [10], [11], [12]. GC-MS methods tend to incorporate a saponification step and measure a combination of both esterified and non-esterified sterols, while LC-MS methods may or may not include base hydrolysis. Using MS methods, a number of exciting discoveries have recently been made. In this short review we will cover the discoveries of most interest to the authors and discuss analytical improvements since our review of analytical methods in 2011 [7].

### 1.1 25-Hydroxycholesterol and immunity

**Fig. 1:**
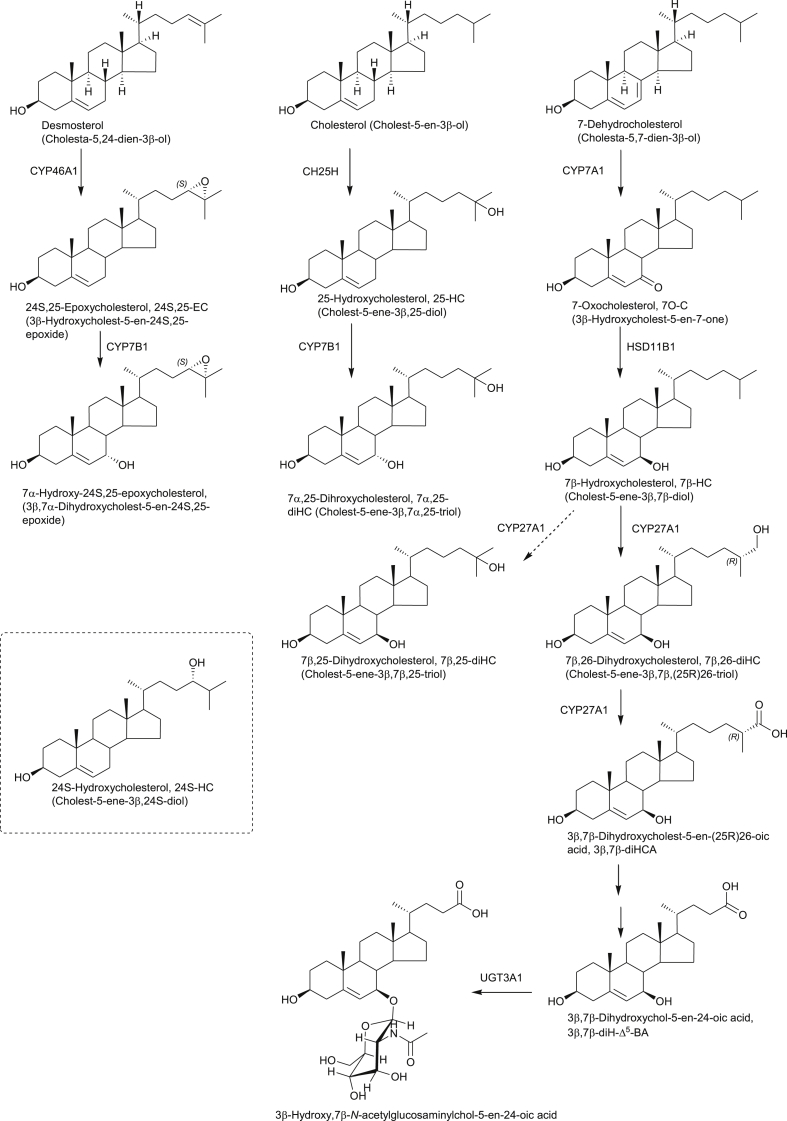
Synthesis and metabolism of repressors of SREBP processing and LXR ligands, 24S,25-EC, 25-HC, of the EBI2 ligand 7α,25-diHC and of the RORγt ligand 7β,26-diHC. Shown in the inset is 24S-HC, a repressor of SREBP processing and an LXR ligand formed from cholesterol by the enzyme CYP46A1. Full stereochemistry is shown only for the initial sterols in the pathways. Systematic names given in parenthesis. Enzymes are indicated where known. A broken arrows indicates a postulated pathway with associated enzyme.

25-Hydroxycholesterol (25-HC) is formed from cholesterol by the enzyme cholesterol 25-hydroxylase (CH25H) and also by other sterol hydroxylases as a minor side-product [13]. It can be 7α-hydroxylated by the enzyme oxysterol 7α-hydroxylase, cytochrome P450 (CYP) 7B1, to give 7α,25-dihydroxycholesterol (7α,25-diHC, Fig. 1). Until recently 25-HC was regarded by analytical biochemists as a minor oxysterol, present at only low levels in plasma (ng/mL), cerebrospinal fluid (CSF, < ng/mL) and most tissues (ng/g). Note, Supplementary Tables S1 and S2 published in Ref. [7] provide lists of literature values for oxysterols measured prior to 2011. However, Diczfalusy et al. found that lipopolysaccharide (LPS; endotoxin) injection to healthy volunteers more than doubled the plasma concentration of 25-HC, while treatment of mouse bone-marrow derived macrophages resulted in a 35-fold increase in expression of *Ch25h* [14]. This finding strongly links 25-HC with the immune system. The oxysterol measurements were made using the classical GC-MS methodology as described by Dzeletovic et al. exploiting isotope dilution mass spectrometry for quantification [1]. About the same time as Diczfalusy et al.'s study was performed, Baumann et al. reported that activation of the Toll-like receptor 4 (TLR4) by Kdo_2_-Lipid A, an LPS substructure with endotoxin activity, resulted in synthesis of 25-HC in mouse intraperitoneal macrophages and an increase in expression of *Ch25h* [15]. Baumann et al. found that treatment of naïve B cells with nanomolar concentrations of 25-HC suppressed interleukin (IL)-2-mediated stimulation of B cell proliferation, repressed activation-induced cytidine deaminase expression, and blocked class switch recombination, leading to markedly decreased immunoglobulin A (IgA) production [15]. They concluded that suppression of IgA class switching in B cells by macrophage-derived 25-HC in response to TLR activation provides a mechanism for local and systemic negative regulation of the adaptive immune response by the innate immune system [15]. In their study, oxysterol measurements were performed by LC-MS using the methodology of McDonald et al. [16]. Dendritic cells (DC) are antigen presenting cells of the mammalian immune system, acting as messengers between the innate and adaptive immune systems. Like macrophages, DCs respond to TLR activation by increasing the expression of *Ch25h* [17]. Park and Scott found that induction of *Ch25h* was mediated by type 1 interferons (IFNs) through the IFN receptor (IFNR) and the JAK/STAT1 pathway [17], [18]. The three studies by Diczfalusy et al., Baumann et al. and Park and Scott identified 25-HC as an important bioactive lipid in the innate and adaptive immune systems and as an important modulator of the immune response to infection [14], [15], [17], [18]. While LPS is an endotoxin, a toxin of the bacterial cell wall, which activates TLRs leading to transcription and translation of cholesterol 25-hydroxylase in macrophages and secretion of 25-HC, viral infection can result in a similar response by macrophages. Blanc et al. showed that upon viral infection or IFN stimulation, *Ch25h* is up-regulated via STAT1 activation leading to 25-HC secretion by macrophages as a potent paracrine inhibitor of many viral infections [19]. In this study Blanc et al. used LC-MS with multistage fragmentation (MS^n^) exploiting “enzyme-assisted derivatisation for sterol analysis” (EADSA, see below) to profile for essentially every known oxysterol [19], [20]. Only 25-HC was secreted by macrophages upon infection or IFN stimulation [19]. In a follow-up study Robertson et al. found that an IFN-regulated microRNA (miRNA), i.e. small non-coding RNA that functions in the post-transcriptional silencing of specific genes, suppressed the cholesterol biosynthesis pathway in activated macrophages as part of the antiviral response [21]. This miRNA, called miR-342-5p, suppressed the pathway at multiple levels: transcriptionally via *SREBF2*; post-transcriptionally via another miRNA, miR-33; and enzymatically via the genes *IDI1* (isopentenyl pyrophosphate isomerase 1) and *SC4MOL* (methylsterol monooxygenase 1). *SREBF2* is the gene encoding sterol regulatory-element binding protein 2 (SREBP2), the master transcription factor regulating the cholesterol synthesis pathway; miR-33 is located within an intron of *SREBF2* and regulates cholesterol homeostasis; while *IDI1* and *SC4MOL* code for enzymes in the cholesterol synthesis pathway. Robertson et al. found that cholesterol and intermediates in its biosynthesis were reduced as part of the macrophage IFN anti-viral response [21]. LC-MS^n^ was used exploiting EADSA to quantify cholesterol and its precursors.

25-HC is a ligand of the liver X receptors (LXR) α and β, members of the nuclear receptor superfamily [22] and a regulator of the processing of SREBP2 to its active form as a transcription factor [23]. Reboldi et al. have suggested that by repressing SREBP2 processing 25-HC reduces *Il1b* transcription and represses IL1-activating inflammasomes [24]. Reboldi et al. concluded that 25-HC is a critical mediator in the negative-feedback pathway of IFN signalling on IL1-family cytokine production and inflammasome activity [24]. This data suggests that reduced synthesis of 25-HC, or its enhanced metabolism, may be involved in the etiology of autoimmune disease.

### 1.2 7α,25-Dihydroxycholesterol is a ligand to the G-Protein coupled receptor - EBI2

7α,25-diHC is usually formed by CYP7B1 mediated hydroxylation of 25-HC. It is present at low levels in both plasma (<ng/mL) and CSF (<ng/mL) [25]. It was found in two independent studies by the pharmaceutical companies Johnson & Johnson [26] and Novartis [27], who published back-to-back papers in Nature in 2011, that 7α,25-diHC is a ligand to the G-protein-coupled receptor (GPCR) EBI2 or GPR183. 7α,25-diHC was found to be a potent agonist of EBI2 and to act as chemoattractant for immune cells expressing EBI2 by directing cell migration in *vitro* and in *vivo*. Similar to EBI2 receptor knockout mice, mice deficient in *Ch25h* failed to position activated B cells within the spleen to the outer follicle and mount a reduced plasma cell response after an immune challenge indicating that the EBI2-oxysterol signalling pathway has an important role in the adaptive immune response [27]. The identification of 7α,25-diHC as the natural ligand for EBI2 was achieved by activity-based purification linked with electrospray ionisation (ESI) - high resolution MS. Accurate mass measurements allowed identification of ions corresponding to [M+H—H_2_O]^+^, [M+H—2H_2_O]^+^, [M+Na]^+^ and [M+CH_3_CO_2_]^-^ of the molecule (M) of formula C_27_H_46_O_3_. Database searches for this formula revealed a dihydroxycholesterol. Of the compounds tested both 7α,25-diHC and 7β,25-diHC were found to activate the EBI2 receptor. NMR analysis of purified material identified 7α,25-diHC as the endogenous compound [27].

### 1.3 7β,26-Dihydroxycholesterol and cholesterol precursors are agonists towards RORγt

The RAR-related orphan receptor gamma t (RORγt) is a nuclear receptor required for generating IL-17-producing CD4(+) Th17 T cells, which are essential in host defence and may play key pathogenic roles in autoimmune diseases. Soroosh et al. using EADSA technology were able to identify 7β,26-dihydroxycholesterol (7β,26-diHC, also commonly called 7β,27-dihydroxycholesterol, Fig. 1) as an agonist of RORγt which could be synthesised by in *vitro* differentiated Th17 cells [28]. Note we use here the systematic nomenclature where hydroxylation of the terminal carbon of cholesterol is at C-26 [29]. This finding still further highlights the involvement of oxysterols in the immune system.

Two other groups have also found sterols to be ligands to RORγt. Santori et al. by overexpression, RNAi, and genetic deletion of metabolic enzymes found a cholesterol biosynthetic intermediate downstream of lanosterol and upstream of zymosterol to activate RORγt [30]. Immunoprecipitation of RORγ followed by LC-MS and GC-MS analysis of bound lipids identified a compound with mass compatible of a hydroxylanosterol, but exact identification was not achieved. In a further study, Hu et al. found the cholesterol precursor desmosterol to be an endogenous RORγ agonist [31].

### 1.4 24S,25-Epoxycholesterol in Parkinson's disease and cholestenoic acids in motor neuron disease

In 2009 Sacchetti et al. reported that an unknown compound promoted ventral midbrain neurogenesis in *vivo* and in human embryonic stem cells through activating the LXR nuclear receptor [32]. In collaboration with Ernest Arenas' group at Karolinska Institute we sought to identify the active molecule. By analysing ventral midbrain from embryonic mouse we found using EADSA and LC-MS^n^ high levels (400 ng/g) of the LXR agonist 24S, 25-epoxycholesterol (24S, 25-EC) and confirmed that this molecule promoted dopaminergic neurogenesis (Fig. 1) [33]. 24S, 25-EC promoted dopaminergic differentiation of embryonic stem cells, suggesting that LXR ligands may contribute to the development of cell replacement therapies for Parkinson's disease (PD).

**Fig. 2:**
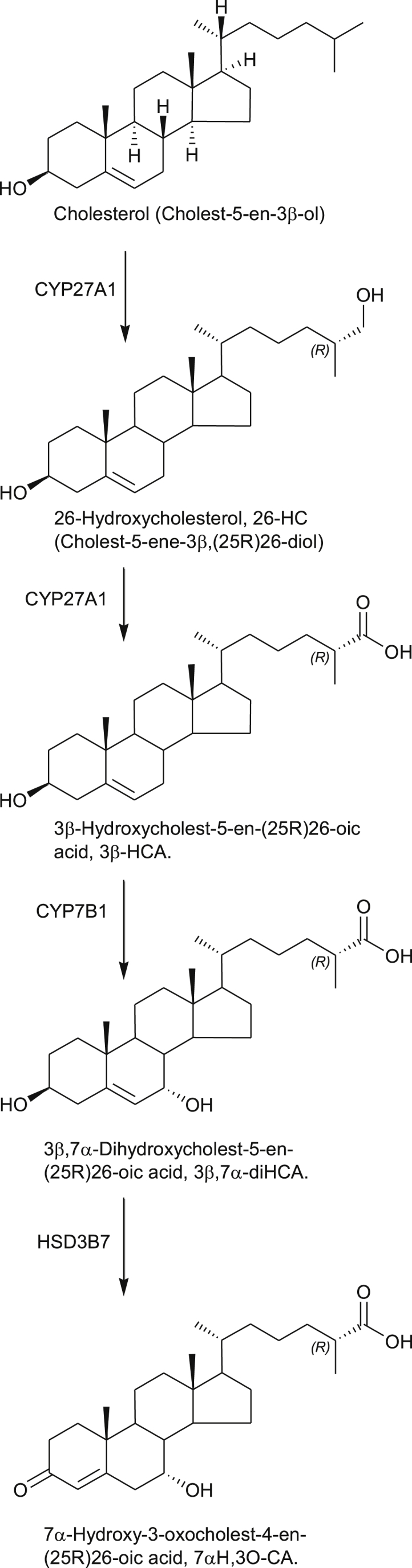
Early steps of the “acidic” bile acid biosynthesis pathway generating 3β-HCA and 3β,7α-diHCA, two cholestenoic acids regulating the survival and death of motor neurons. Full stereochemistry is shown only for the initial sterol in the pathway. Systematic names given in parenthesis. Enzymes are indicated where known.

Not only are LXR ligands likely to be important in dopaminergic neurogenesis, Andersson et al. have shown that deletion of LXRβ, the predominant isoform in the central nervous system (CNS), results in adult-onset motor neuron degeneration [34]. What might then be the LXR ligand which is protective towards motor neurons? Clues to the answer to this question come from the disorder called hereditary spastic paresis type 5 (SPG5) which is a genetic disorder which presents with motor neuron loss. From analysis of plasma and CSF of patients with SPG5 we found that levels of the cholesterol metabolite 3β,7α-dihydroxycholest-5-en-(25R)26-oic acid (3β,7α-diHCA, Fig. 2) are reduced (in plasma from 39.40 ± 3.95 ng/mL, mean ± SE, in controls to 5.21 ± 3.09 ng/mL in SPG5 patients and in CSF from 2.12 ± 0.39 ng/mL in controls to 0.47 ± 0.44 ng/mL in SPG5 patients) [25]. This is a consequence of mutations in the *CYP7B1* gene. We were able to show that 3β,7α-diHCA promoted motor neuron survival in an LXR-dependent manner, and that another metabolite elevated in SPG5, 3β-hydroxycholest-5-en-(25R)26-oic acid (3β-HCA, elevated in plasma to 368.40 ± 65.27 ng/mL from 81.12 ± 4.31 ng/mL in controls, and in CSF to 20.14 ± 2.76 ng/mL from 0.96 ± 0.10 ng/mL in controls), was toxic towards motor neurons. Our results indicate that specific cholestenoic acids selectively work on motor neurons, to regulate the balance between survival and death [25].

### 1.5 24S-Hydroxycholesterol in the CNS and other tissue

24S-Hydroxycholesterol (24S-HC) is synthesised in neurons of the CNS from cholesterol via the enzyme CYP46A1 [13]. It has been suggested as a potential marker of neurodegeneration [3]. Neurodegeneration can be associated with increased levels of 24S-HC and 26-hydroxycholesterol (26-HC, commonly called 27-hydroxycholesterol, 27-HC) in CSF, probably due to the release of oxysterols from dying neuronal cells or in the case of 26-HC due to defects in the blood brain barrier (BBB). On the other hand concentrations of 24S-HC in the circulation can reflect the number of biologically active neurons and CNS cholesterol turnover [3]. Recently, Björkhem et al. have found using classical GC-MS methods that concentrations of 24S-HC in CSF of PD patients correlate with the duration of the disease but plasma levels are within the normal range [35]. In a similar study investigating the effect of natalizumab treatment on oxysterol levels in patients with relapsing remitting multiple sclerosis, again using classical GC-MS methods, Novakova et al. found that drug treatment reduced the levels of 24-HC and 26-HC in CSF following treatment. This was explained by reduced neuronal damage and restored BBB integrity upon treatment [36].

In 2006 David Russell's group at University of Texas Southwestern Medical Center showed that knock out of *Cyp46a1* in mice resulted in severe deficiencies in spatial, associative and motor learning [37]. It was suggested that a continuous formation of 24S-HC is required for a sufficient flux through the mevalonate pathway for formation of adequate levels of isoprenoids, such as geranylgeraniol, critical for memory function [37]. Björkhem's group in Sweden have now generated a homozygous mouse overexpressing human HA-tagged CYP46A1 [38]. Unsurprisingly, plasma levels of 24-HC, as determined by classical GC-MS methods, were elevated 6–7 fold and also elevated in brain from 31 ± 1 ng/mg tissue in wild type (wt) to 40 ± 0 ng/mg tissue in the homozygous overexpressing animals. Lanosterol, a marker of cholesterol biosynthesis, was significantly increased in brain of the homozygous overexpressing animals, but cholesterol in brain was not significantly different between wt and transgenic animals [38]. When tested in the Morris water maze, 15 month old female animals showed an improvement in spatial memory. These results indicate that flux through the mevalonate pathway is important for cognitive functions in brain.

Mounting evidence indicates that CYP46A1 is expressed in cells other than neurons. In a very recent study exploiting EADSA for oxysterol analysis Soncini et al. found 24S-HC increased in the pancrease of a mouse model of pancreatic neuroendocrine tumour (pNET) development [39]. They showed that hypoxia inducible factor (HIF-1α) controls the over expression of CYP46A1 in the mouse model of pNET which ultimately leads to induction of the angiogenic switch through positioning of proangiogenic neutrophils in proximity of CYP46A1^+^ islets [39].

### 1.6 Oxysterols and the Hedgehog signalling pathway

**Fig. 3:**
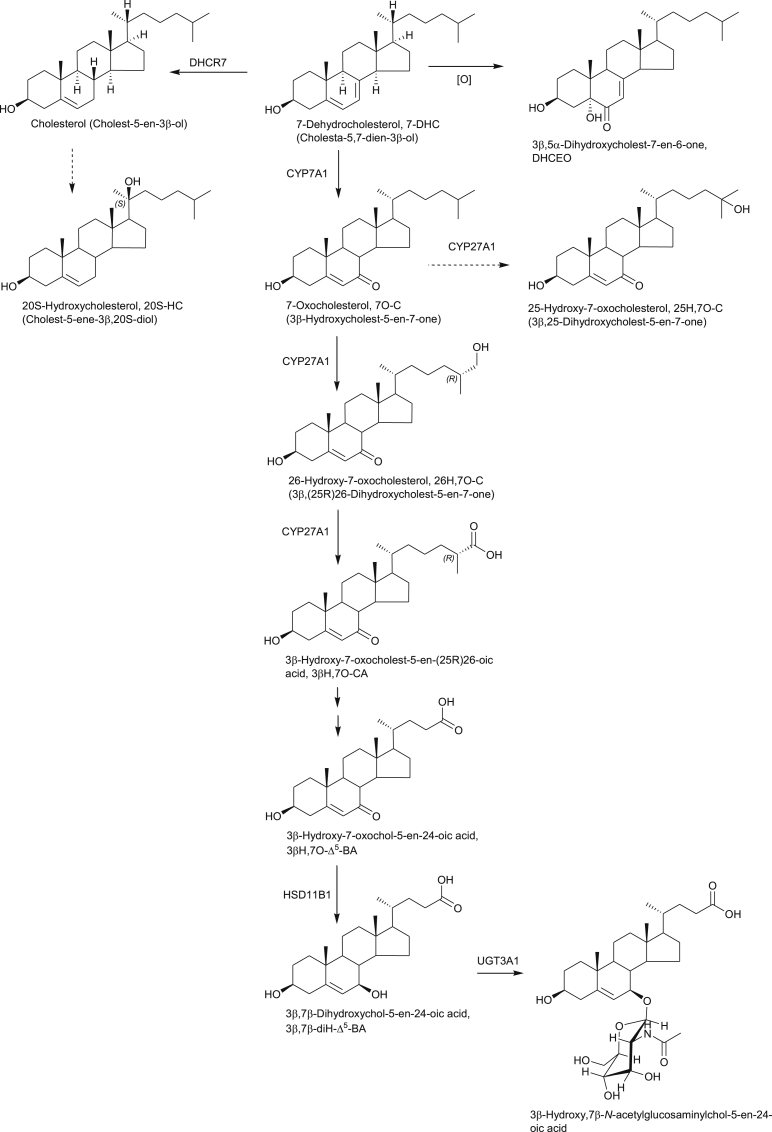
Synthesis and metabolism of oxysterol modulators of Hh signalling. Some of the oxysterols are prevalent in patients suffering from NP-C disease or SLOS. Full stereochemistry is shown only for the initial sterols in the pathways. Systematic names given in parenthesis. Enzymes are indicated where known. Broken arrows indicate postulated pathway and enzyme.

In the sections above, the involvement of oxysterols in the immune and nervous systems has been discussed. Molecules with similar, but not exactly the same structures are also of great importance in the Hedgehog (Hh) signalling pathway which controls numerous aspects of embryonic patterning and regeneration of postembryonic tissues [40], [41], [42]. Insufficient Hh signalling can result in a range of birth defects, and inappropriate pathway activity is linked to human malignancies such as basal cell carcinoma and medulloblastoma. The pathway is activated by binding of the cholesterol modified Hh protein to its receptor protein Patched 1 (Ptch1). This releases inhibition on the oncoprotein Smoothened (Smo) by Ptch1, with resultant pathway activation. The Hh pathway is modulated through Smo by a number of mechanisms, (i) there is a requirement for cellular sterols, (ii) the pathway is activated by specific oxysterols and (iii) the pathway may be inactivated or activated by binding of ligands at the so called cyclopamine binding pocket. Smo is a seven transmembrane protein with an extracellular cysteine rich domain (CRD). It belongs to the GPCR family and shows structural similarity to the Frizzled (Fz) Wnt receptors. Cyclopamine is a plant alkaloid with an A/B ring system similar to that found in cholesterol, and its binding pocket is within the transmembrane domain (TMD) of Smo. On the other hand, oxysterols which activate Smo bind to the extracellular CRD [40], [41], [42]. Very recently, Byrne et al. have shown that the putative oxysterol binding site in the CRD of Smo can also bind cholesterol which occupies the CRD groove stabilising a resting conformation poised to respond to Hh signals. Byrne et al. suggested that oxysterols can displace cholesterol and produce a conformational change that leads to Smo activation [43]. Interestingly, the CRD of the Niemann-Pick C1 (NPC1) protein also binds cholesterol (see below). 20S-Hydroxycholesterol (20S-HC, Fig. 3) has been known for some time as an activator of Hh signalling through interacting with Smo. This is believed to be at the CRD. However, 20S-HC has only once been reported as an endogenous molecule in animals and this finding has not been confirmed subsequently [44], [45]. Smith-Lemli-Opitz syndrome (SLOS, see below) is an autosomal recessive disorder which can present with features compatible with disordered Hh signalling [46]. It is characterised by deficiency in the enzyme 7-dehydrocholesterol reductase (DHCR7), leading to elevated concentrations of 7-dehydrocholesterol (7-DHC) and 7-oxocholesterol (7O-C) in the circulation. 7O-C can be further metabolised to both 26-hydroxy-7-oxocholesterol (26H,7O-C, commonly called 27-hydroxy-7-oxocholesterol) and 25-hydroxy-7-oxocholesterol (25H,7O-C, Fig. 3) [47]. Myers et al. have shown that both 26H,7O-C and 25H,7O-C activate the Hh signalling pathway by binding to the CRD of Smo [40]. In contrast to these two dihydroxycholest-5-en-7-one sterols, another cholesterol derivative 3β,5α-dihydroxycholest-7-en-6-one (DHCEO, Fig. 3) also derived from 7-DHC, but by free radical oxidation, has been found to be an inhibitor of Hh signalling, apparently by a mechanism distinct from that employed by side-chain oxysterols [48]. As over activity of the Hh pathway is linked to some cancers, and inhibitors of Smo which act by binding to the cyclopamine pocket of the TMD have been approved as anti-cancer drugs, the discovery of DHCEO as an inhibitor of Hh signalling may provide a new lead for anticancer drug development. In this regard we have found that 3β-hydroxy-7-oxocholest-5-en-(25R)26-oic acid (3βH,7O-CA), a metabolite of 26H, 7O-C, is an inhibitor of Hh signalling, presumably through interaction with Smo at the CRD binding pocket [47].

### 1.7 Oxysterols and breast cancer

**Fig. 4:**
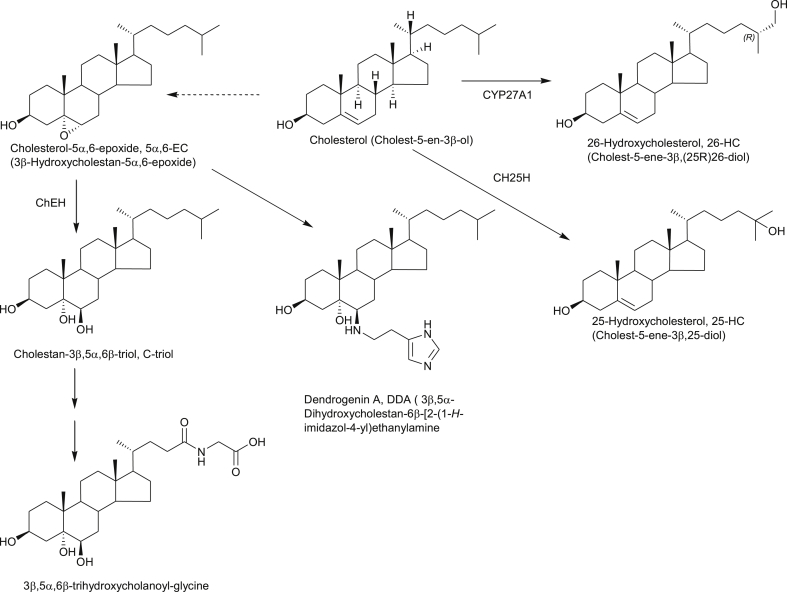
Oxysterols implicated in the cause of cancer and metabolism of 5,6-EC. Full stereochemistry is shown only for the initial sterol in the pathways. Systematic names given in parenthesis. Enzymes are indicated where known. Broken arrows indicate postulated pathway.

Two papers published in November 2013 describe a link between 26-HC and breast cancer [49], [50]. Nelson et al. found that 26-HC promoted tumour growth and metastasis in mouse models of breast cancer by acting via the estrogen receptor α (ERα) and LXR, respectively [49]. Aggressive breast cancers were found to express high levels of CYP27A1, the enzyme converting cholesterol to 26-HC (Fig. 4), indicating that 26-HC within tumours may contribute to the disease. Nelson et al. speculated that lowering circulating levels of 26-HC or inhibiting its synthesis by CYP27A1 may offer a strategy to prevent or treat breast cancer [49]. In the second paper, Wu et al., found that 26-HC stimulates MCF-7 cell xenograft growth in mice [50]. In ER+ breast cancer patients the 26-HC content in normal breast tissue was increased compared to that in cancer-free controls, and tumour 26-HC content was further elevated [50]. In this second study oxysterol measurements were made by LC-MS using the methodology outlined in Ref. [51]. In a more recent study, Simigdala et al. found by global gene expression studies that the cholesterol biosynthesis pathway was up-regulated in ER+ long-term estrogen deprivation (LTED) cell lines but not ER– LTED cell lines [52]. ER+ breast cancer cells express ERα, while ER-cells do not. LC-MS analysis, exploiting EADSA, revealed elevated levels of 25-HC in MCF7 LTED cells. Further studies showed that exogenous 25-HC or 26-HC increased ER-mediated transcription and expression of endogenous estrogen-regulated genes [52]. The authors concluded that elevated levels of 25-HC or 26-HC may explain resistance to endocrine therapy in ER+ breast cancer patients and has potential as a therapeutic target [52].

While 25-HC and 26-HC are implicated in the cause of breast cancer, another oxysterol, dendrogenin A (DDA) may be an important tumour suppressor. DDA is formed by the stereo specific nucleophilic addition of histamine to cholesterol-5α,6-epoxide (5α,6-EC, Fig. 4) and Poirot and colleagues have shown that DDA causes cancer cell differentiation and has anticancer properties both in *vitro* and in *vivo* [53]. 5α,6-EC is usually thought of as a non-enzymatic product of cholesterol, but there is some evidence for its enzymatic formation [54], [55]. 5α,6-EC and its epimer, 5β,6-EC, are both ligands to the microsomal antiestrogen binding site (AEBS), a hetero-oligomeric protein to which tamoxifen, a drug which binds to the ERα receptor, also binds. The AEBS shows cholesterol-5,6-epoxide hydroxylase (ChEH) activity, converting 5,6-EC to cholestane-3β,5α,6β-triol (C-triol) and inhibition of ChEH activity by e.g. tamoxifen, induces cancer cell re-differentiation through accumulation of the DDA precursor 5α,6-EC and its epimer [53]. Interestingly, the AEBS is made up of two subunits, 3β-hydroxysteroid-Δ^8^,Δ^7^-isomerase (D8D7I) and DHCR7, two enzymes of the cholesterol biosynthesis pathway. Using LC with nano-ESI-MS de Medina et al. characterised DDA in mouse brain, the multiple nitrogens on the histamine tail facilitating ionisation, and determined reduced concentrations in breast tumours compared to controls [53]. Furthermore, DDA was not present in breast cancer or melanoma cell lines. It seems likely that the anticancer action of DDA is through the immune system as DDA treatment was found to stimulate T lymphocytes and dendritic cells infiltration of engrafted tumours in mice [53].

### 1.8 Niemann-Pick type C disease

Niemann-Pick type C (NP-C) disease is characterised by cholesterol accumulation in lysosomes and aberrant regulation of cholesterol homeostasis. It is an autosomal recessive disorder with progressive neurodegeneration leading to death in early childhood. Two proteins implicated in NP-C are the 13-transmembrane NPC1 and the secreted NPC2. Interestingly, NPC1 shares significant homology with Ptch1 [56] and also has a lipid binding protein fold conserved within N-terminal CRDs of Fz receptors, which include Smo [57]. In 2001 Alvelius et al. found high levels of bile acids with a 3β-hydroxy-5-ene structure carrying a 7-oxo or 7β-hydroxy group in urine and plasma from an NP-C patient by exploiting GC-MS and nano-ESI-MS methods (Fig. 3) [58]. Since then, 7O-C, a precursor of both the 7-oxo and 7β-hydroxy acids has been proposed as a plasma biomarker for NP-C disease [59]. Formation of the former acid proceeds through 26H,7O-C (Fig. 3), which is a ligand to Smo as discussed above, while the latter acid can also be generated through the same pathway but also via 7β,26-diHC (Fig. 1). The linkage between NPC1, Ptch and Smo proteins and the NP-C and SLOS metabolite 26H,7O-C is very intriguing.

Besides 7O-C, C-triol has been shown to be a biomarker for NP-C, and methods have been developed based on GC-MS [59] and LC-MS with derivatisation to dimethylglycine esters for its analysis (see below) [12], [60]. Using this derivatisation method and LC-MS, Klinke et al. demonstrated that not only NP-C, but also NP-A and NP-B, which have a similar clinical picture to NP-C, show elevated levels of 7O-C and C-triol in plasma [61]. A danger of making a clinical diagnosis based on 7O-C and C-triol is that the former is a direct autoxidation product of cholesterol, while the latter can be formed by hydrolysis of 5,6-EC, which may similarly be formed via autoxidation. Autoxidation of cholesterol can occur during sample handling in air [62].

To avoid the problem of autoxidation, Mazzacuva et al. have developed an LC-MS method utilising negative-ion ESI for the analysis of the conjugated bile acids 3β-hydroxy,7β-*N-*acetylglucosaminylchol-5-enoic acid (Fig. 3) and 3β,5α,6β-trihydroxycholanoyl-glycine (Fig. 4) both of which accumulate in NP-C plasma and are enzymatically derived metabolic products of 7O-C and C-triol, respectively [63]. As the GlcNAc transferase, UGT3A1, is commonly mutated and inactive, 3β,5α,6β-trihydroxycholanoyl-glycine was concluded to be the better marker of NP-C [63]. A second piece of evidence for this conclusion is that we have recently shown that 3β-hydroxy, 7β-*N-*acetylglucosaminylchol-5-enoic acid is also a metabolic product found in patients with SLOS [47].

### 1.9 Smith-Lemli-Opitz-syndrome

**Fig. 5:**
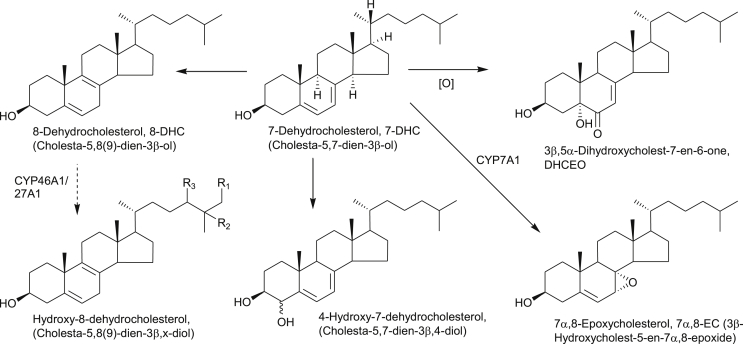
Some primary products of 7-DHC oxidation found in patients with SLOS. Further metabolic products are shown in Fig. 3. Full stereochemistry is shown only for the initial sterol in the pathways. Systematic names given in parenthesis. Enzymes are indicated where known. Broken arrows indicate postulated pathway.

**Fig. 6:**
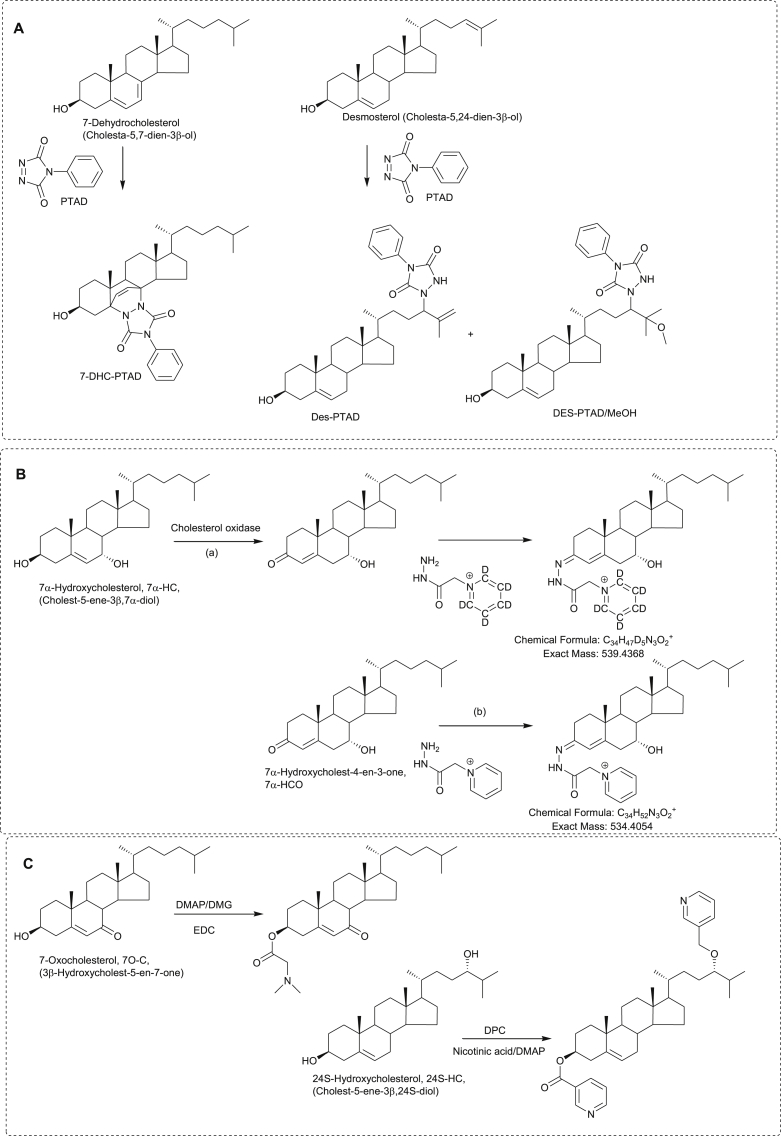
Derivatisation reactions for sterol and oxysterol analysis by LC-MS (A) Derivatisation with PTAD. (B) Derivatisation with GP reagent. (C) Left hand side, derivatisation with dimethylglycine; right hand side, derivatisation with nicotinic acid. Abbreviations: DMAP, 4-(dimethylamino)pyridine; DMG, *N,N*-dimethylglycine; EDC, 1-ethyl-3-(3-dimethyl-aminopropyl) carbodiimide; DPC, *N,N*-diisopropylcarbodiimide.

SLOS is a severe autosomal recessive disorder resulting from defects in the cholesterol synthesising enzyme DHCR7, leading to a build-up in the cholesterol precursor 7-DHC and its isomer 8-DHC in tissues and blood plasma (Fig. 3, Fig. 5) [46]. The phenotypic spectrum of SLOS is very broad. Severe cases die in utero, mild cases show only minor physical, learning and behavioural problems. Limb abnormalities are, however, common in SLOS patients [46]. For diagnosis, levels of 7-DHC are usually measured by GC-MS, however, recently Liu et al. have developed an LC-MS method based on derivatisation with 4-phenyl-1,2,4-triazoline-3,5-dione (PTAD, Fig. 6) through a Diels-Alder cycloaddition which is applicable for analysis of sterols with a 5,7-diene structure as in 7-DHC (see below) [9].

Using LC-MS^n^ incorporating EADSA technology we recently profiled oxysterols derived from 7-DHC and 8-DHC in human plasma [64]. We found a novel hydroxy-8-dehydrocholesterol, where hydroxylation was either at C-24 or C-25 [64], and also identified the known metabolites 26-hydroxy-8-dehydrocholesterol [65], 4-hydroxy-7-dehydrocholesterol [66], 7α,8-epoxycholesterol (7α,8-EC) [67] and DHCEO [68] (Fig. 5). None of these metabolites were detected in control plasma at quantifiable levels (0.5 ng/mL) [64]. Importantly, this was the first identification of DHCEO in human plasma, although it has been found to be a major product of 7-DHC free radical oxidation in tissues and fluids from a rat model of SLOS [69]. We also found elevated levels of 7O-C in SLOS plasma [67], in agreement with an earlier study by Liu et al. [66]. Shinkyo et al. have shown that 7O-C can be formed from 7-DHC by CYP7A1 (Fig. 3), this explains its high level in SLOS plasma [70], 7α,8-EC is formed as a side-product to this reaction (Fig. 5) [67], [70]. In a very recent study, using LC-MS^n^ and EADSA, we have found that 7O-C can be further metabolised in SLOS patients to 26H,7O-C and also 25H,7O-C, and that the former compound can be metabolised further to 3βH,7O-CA (Fig. 3) [47]. Importantly, SLOS is a genetic disease that phenocopies aberrant Hh signalling and 26H,7O-C and 25H,7O-C are activators of Hh signalling [40] while 3β,7O-CA is an inhibitor [47].

## 2 Analytical improvements

Since our review of “Oxysterol Metabolomes” in 2011 [7], there have been a number of improvements in analytical methods for the analysis of oxysterols and related compounds. McDonald and colleagues in Dallas have refined their LC-MS methods which are now applicable for analysis of more than 50 sterols and oxysterols from only 200 μL of plasma with detection limits of 1 ng/mL [8], [51]. In an effort to improve sensitivity, there has been a revival of interest of exploiting derivatisation chemistry to enhance LC-MS analysis of oxysterols. Our preference is for EADSA where oxysterols are first oxidised at C-3 to a 3-oxosteroid, usually by conversion of the 3β-hydroxy group to a 3-oxo with cholesterol oxidase, then reaction with the Girard P (GP) reagent to enhance ESI signal and direct fragmentation upon MS/MS or MS^n^ (Fig. 6) [11], [71]. A disadvantage of this method is that it requires duplicate analysis, (a) with, and (b) without, addition of cholesterol oxidase to differentiate between compounds that naturally contain a 3-oxo group and those that naturally contain a 3β-hydroxy group (see Fig. 6B). We have now introduced a [^2^H_5_]GP reagent which allows the (a) and (b) fractions to be analysed simultaneously by LC-MS^n^. Derivatisation with Girard reagents offers improved specificity and sensitivity and this has been exploited by other groups using mainly the Girard T (GT) reagent rather than GP [28], [39], [52]. Impressively, using EADSA and GT derivatisation Roberg-Larsen et al. were able to show that 26-HC is elevated in exosomes from an ER+ breast cancer cell line derived from only 200,000 cells [72]. This group use low flow-rate chromatography with ESI to enhance sensitivity and incorporate on-line solid-phase extraction for sample clean-up. Of the various other derivatisation methods, the one developed by Jiang et al. [73] where the free hydroxy group(s) is derivatised with *N,N-*dimethylglycine (DMG) has become popular for identification of NP-C disease (Fig. 6C) [12], [61]. This method suffers from an inherent lack of specificity on account of the prevalence of hydroxy-containing molecules in nature. An alternative derivatisation targeted at hydroxy groups is derivatisation to picolinic acid esters [74] or nicotinic acid esters [10]. Sidhu et al. have exploited the latter derivatisation to analyse oxysterols in CSF and plasma [10] (Fig. 6C).

A new derivatisation for sterols in general is reaction with PTAD (Fig. 6A). Although this derivatisation is well known for vitamins D analysis, Liu et al. have shown that it can be used for unsaturated sterols in general [9]. By careful choice of conditions, the reaction can be “tuned” to favour reaction with B-ring unsaturated sterols like cholesterol via an “ene” reaction, or with side-chain double bonds as in desmosterol, also via an “ene” reaction, or with conjugated dienes like 7-DHC in a Diels-Alder reaction. The PTAD derivatisation offers improvement in sensitivity and also provides a route to stabilising reactive dienes [9].

Matrix-assisted laser desorption/ionisation (MALDI)-MS imaging is an exciting technology for determining the location of molecules in a tissue. By coating tissue, e.g. 10 μm thick coronal section of rodent brain, with matrix and mounting the tissue on a stage then moving the stage relative to the laser, it is possible to record mass spectra at discrete pixels and reconstruct an image of a specific ion over the tissue. However, as in all MS, only molecules that can be ionised will be observed. Cobice et al. have cleverly exploited GT derivatisation to image 7O-C in mouse brain [75]. In the absence of GT derivatisation any signal from 7O-C is hidden by back-ground noise. Cobice et al. found that by using GT derivatisation a significant increase in 7O-C signal was observed in brain sections from hydroxysteroid dehydrogenase (HSD) 11B1 deficient mice [75]. HSD11B1 is the enzyme which converts 7O-C to 7β-HC and in its absence 7O-C accumulates.

## 3 Conclusions

When the authors joined the field of oxysterol analysis around the turn of the century, oxysterols and cholestenoic acids were mostly considered as uninteresting intermediates of cholesterol metabolism. Some oxysterols had been shown to have biological activity, but the most studied oxysterol, 25-HC, was detected at low levels in biological samples, and was often suspected to be an artefact of sample handling [62]. Despite notable exceptions [3], [62], [76], [77], [78], [79] few researchers were interested in the biochemistry of oxysterols. However, in the last 5–10 years we have seen a paradigm shift, with a huge surge of interest in oxysterol biochemistry. There have been major developments in MS technology and methods for oxysterol analysis, but problems still are evident. Oxysterols can be formed from cholesterol in air, so there is always the danger of their artefactual formation through sample handling. There are a huge number of isomeric oxysterols. We now know that not only is 7α,26-diHC a biologically relevant molecule on the pathway to bile acid biosynthesis, but is its epimer, 7β,26-diHC, is also biologically relevant. Similarly, both 24S-HC and 24R-HC can be found in biological samples [80]. The analyst must be aware of the huge potential for isomeric structures or important molecules will be missed or misidentified and if chromatographic separation is inadequate quantitation will be inaccurate. Despite these reservations the future for cholesterolomics is bright.
